# Assessing the Completeness of Safety Reporting in Clinical Trials of Total Knee Arthroplasty: A Registry-Publication Comparison Study

**DOI:** 10.2106/JBJS.OA.25.00346

**Published:** 2026-02-11

**Authors:** Nicholas Camasso, Garrett Herring, Kellen Keefer, Ryan Sherry, Ahmed Elghzali, Tag Harris, Daniel Archer, Alicia Ito Ford, Matt Vassar

**Affiliations:** 1 Office of Medical Student Research, Oklahoma State University Center for Health Sciences, Tulsa, Oklahoma; 2 Department of Psychiatry and Behavioral Sciences, Oklahoma State University Center for Health Sciences, Tulsa, Oklahoma

## Abstract

**Background:**

Total knee arthroplasty (TKA) clinical trials inform surgical decisions by reporting adverse events (AEs), including serious adverse events (SAEs), other adverse events (OAEs), and deaths. However, concerns persist regarding discrepancies in AE reporting between trial registries, such as ClinicalTrials.gov, and peer-reviewed publications, even after the implementation of Food and Drug Administration Amendments Act Section 801 and the Final Rule of 2017, laws introduced to mitigate selective reporting and enhance public transparency.

**Methods:**

We conducted a systematic registry-to-publication comparison of 92 TKA-focused clinical trials with posted results on ClinicalTrials.gov between 2009 and 2024. Data on SAEs, OAEs, and deaths were extracted from registries and matched peer-reviewed publications using a pre-registered protocol. Descriptive statistics were used to evaluate discrepancies and trends over time. Regression analysis was used to assess the impact of the Final Rule and variables associated with changes in reporting score.

**Results:**

AE reporting was consistently more complete in ClinicalTrials.gov entries than in publications. SAE count mismatches were present in 95% of trials, and mortality data were omitted from 87% of pre-Final Rule Applicable Clinical Trial (ACT) publications. Post-Final Rule trials continued to underreport SAEs and deaths in publications, with no significant improvement in reporting completeness. Only 15% of trials listed AEs as formal outcomes in registries, and 66% of post-Final Rule ACTs omitted SAE reporting in the publication.

**Conclusion:**

Despite regulatory mandates, AE reporting in TKA trials remains inconsistent and incomplete across publications. These discrepancies risk undermining surgical decision-making and evidence-based guidelines. Enhanced enforcement, editorial accountability, and stricter adherence to reporting standards such as Consolidated Standards of Reporting Trials Harms are necessary to improve transparency and patient safety in orthopaedic research.

**Level of Evidence:**

Level II. See Instructions for Authors for a complete description of levels of evidence.

## Introduction

Total knee arthroplasty (TKA) has become a highly customizable procedure; surgeons determine their strategic approach not solely on functional outcomes but also on the risk of adverse events (AEs) such as infection, thromboembolism, and pain^1,2^. Accurate AE data is critical to inform decision making and patient counseling.

Underreporting of AEs is a widespread problem in medicine. Previous studies in oncology and multiple myeloma trials, for example, have highlighted the omission of AE data in their publications, and in orthopaedics, randomized controlled trials (RCTs) informing American Academy of Orthopaedic Surgeons (AAOS) Clinical Practice Guidelines (CPG) have shown low adherence to the Consolidated Standards of Reporting Trials (CONSORT) Harms checklist.^3-8^

Although discrepancies between trial registries and their associated publications have been described, this issue has not been systematically explored in TKA trials^9-13^. Despite Food and Drug Administration Amendments Act 801 and the Final Rule requiring AE reporting to ClinicalTrials.gov,^14,15^ it is unclear how faithfully this information is represented in peer-reviewed TKA publications. Our primary objective was to assess discrepancies in reporting of serious adverse events (SAEs), other adverse events (OAEs), and mortality between ClinicalTrials.gov and corresponding TKA RCTs. As a secondary objective, we evaluated whether reporting completeness improved after the 2017 Final Rule to examine the impact of mandated reporting requirements on AE transparency.

## Methods

### Study Design

We conducted a registry-to-publication comparison study to evaluate AE reporting in TKA clinical trials and followed the Preferred Reporting Items for Systematic Reviews and Meta-Analyses (PRISMA) guidelines^16^. Our methodology was adapted from previous studies^17-19^. The Oklahoma State University Center for Health Sciences Institutional Review Board determined this study did not meet the definition of human subjects research and was exempt from further oversight under 45 Code of Federal Regulations (CFR) 46.102(d) and (f)^20^. The protocol was preregistered with PROSPERO,^21^ and all study materials (search strategy, included trials, raw data, extraction forms, analysis scripts) are posted on Open Science Framework (OSF)^22^.

### Search Strategy

On June 28, 2025, we searched ClinicalTrials.gov for Phase 2 to 4 or “not applicable” trials with posted results and start dates between September 27, 2009, and December 31, 2024, to align with the Final Rule (42 CFR §11.44)^23^. Only trials with publicly available results were included.

To maximize precision, we constructed a TKA-specific query using quoted phrases and Boolean operators, avoiding reliance on ClinicalTrials.gov's automatic synonym expansion^24^. Two reviewers (N.C., G.H.) independently identified corresponding publications linked on ClinicalTrials.gov, supplemented with PubMed (via NCBI) and Google Scholar searches.

ClinicalTrials.gov was used as the sole AE data source because of its standardized, FDAAA 801-mandated format. Registries like the European Union Clinical Trials Register and World Health Organization International Clinical Trials Registry Platform were excluded for inconsistent harms reporting^25,26^.

### Eligibility Criteria

**Fig. 1:**
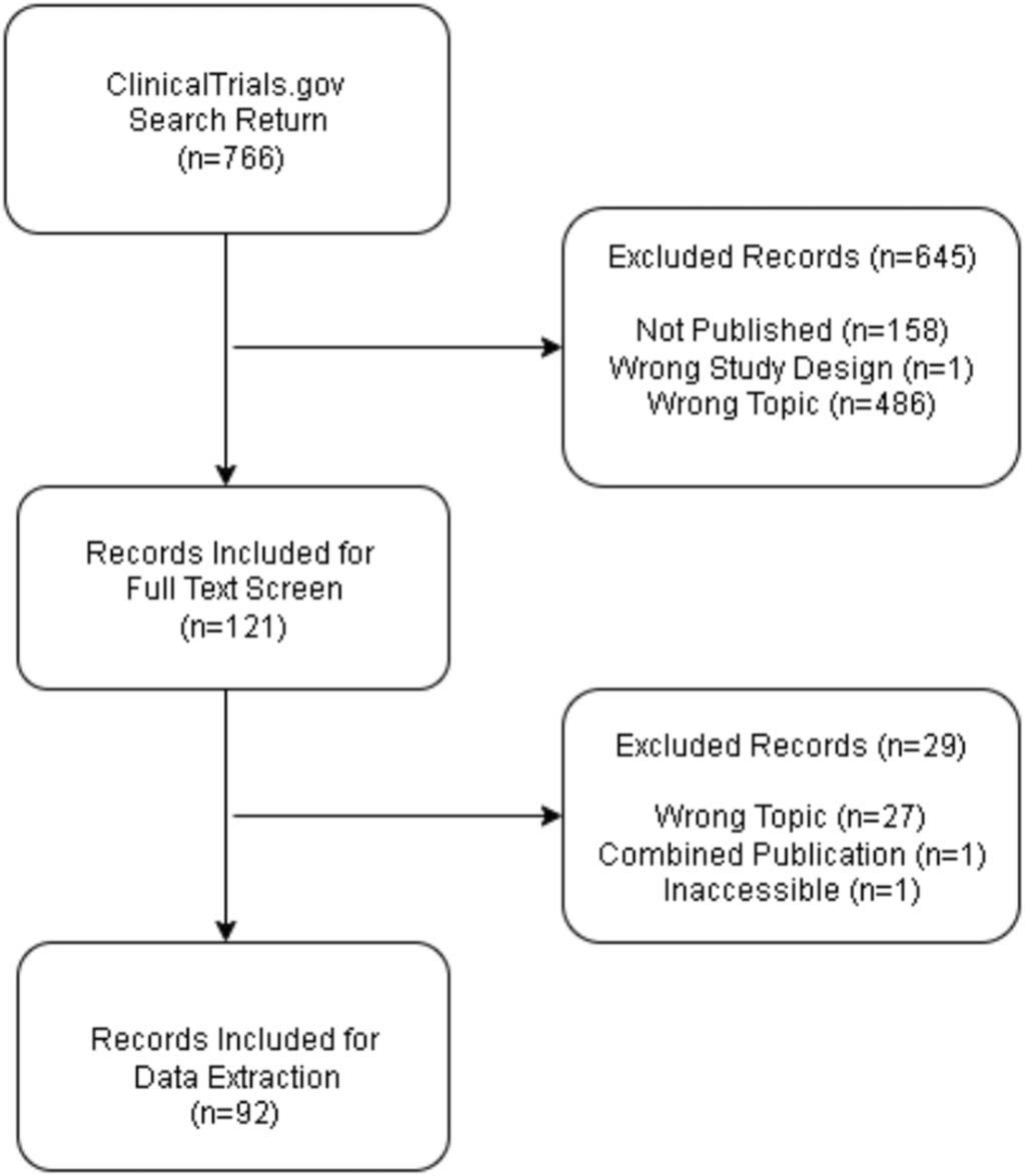
Of 766 records identified through the ClinicalTrials.gov search, 92 trials met all inclusion criteria. Records were excluded because of lacking a peer-reviewed publication (n = 158), having the wrong study design (n = 1), being centered on the wrong topic (n = 513), having combined publications (n = 1), and having inaccessible articles (n = 1). The diagram illustrates the number of records screened, excluded, and included for full-text review and data extraction.

Eligible studies were registered on ClinicalTrials.gov with posted results and evaluated a primary TKA intervention. We excluded those that were observational studies, lacking a publication, or enrolling individuals younger than 18 years of age. A PRISMA flow diagram (Fig. 1) summarizes study selection.

### Dataset Criteria and Selection

We included both FDA-regulated trials subject to mandatory reporting and nonregulated trials, without such requirements, informative of AE reporting practices. Because applicable clinical trial (ACT) status is not publicly designated, we inferred eligibility using the ACT checklist, defined by 42 CFR §11.22(b)^27^. Trials started on or after January 18, 2017, were assessed using the checklist; those beginning before this date were assessed with additional clarification from the National Institute of Health's (NIH) 2009 guidance document on ACT criteria^28^. Eligible ACTs were interventional FDA-regulated drug (Phase 2-4) or device (nonfeasibility) studies with a US nexus (i.e., a US site, exported product, or conducted under an Investigational New Drug/Investigational Device Exemption)^23^. Inclusion also required a corresponding English language publication.

The non–FDA-regulated data set included trials exempt from reporting under 42 CFR Part 11 and expanded access trials (21 U.S.C. §360bbb), which are exempt from both federal registration and reporting mandates^29^.

### General Data Extraction

Two reviewers (N.C., G.H.) independently extracted registry and publication data using a standardized Google Form, with disagreements resolved by consensus with a third reviewer (K.K.). Reviewers completed calibration on 10 pilot studies before extraction.

Extracted trial data included National Clinical Trial (NCT) identifier, key trial dates, phase, sponsor, and funding source. Harms data included counts of SAEs, OAEs, AE-related withdrawals, and all-cause mortality. We documented discrepancies between registry and publication reporting (e.g., reporting thresholds and omission of harms) and noted whether publications referenced the CONSORT Harms extension or used Clavien-Dindo classification. AEs were defined per 42 CFR §11.10(a) as any unfavorable medical occurrence during the trial, regardless of causality^23^. SAE classifications were taken as reported in both sources, with no verification or reclassification by the study team. SAE definitions and reporting practices varied across trials, and discrepancies may reflect heterogeneity in how investigators applied SAE criteria. Terms such as “side effects” or “complications” were treated as AEs. Reporting ambiguities and context were noted.

For cross-trial comparison, we extracted sponsor-defined AE and SAE categories reported in ClinicalTrials.gov and mapped publication-reported events to these categories when possible. Because many publications did not differentiate AEs from SAEs, all reported events were treated as AEs for consistency. Initial attempts to create more granular, TKA-specific AE categories were limited by substantial variability in registry terminology; therefore, analyses relied on the predefined sponsor-selected categories listed within the registry.

We did not assess AE definition consistency or evaluate causality of deaths. In our analysis, all events were analyzed as reported.

### Data Analysis

Descriptive statistics (frequencies, percentages, medians with IQRs) were used to characterize trial attributes (e.g., phase, sponsor, enrollment) and summarize AE reporting patterns. Bland-Altman plots visualized SAE concordance; funnel plots (proportion of SAEs vs. enrollment) were inspected for asymmetry. Chi-square tests were used to compare categorical variables (e.g., presence/absence of AE reporting) across sponsor types and study phases; the Mann-Whitney *U* test was applied to non-normal outcomes like SAE counts.

We performed unadjusted and adjusted linear regression analyses to identify trial-level predictors of AE reporting, treating categorical predictors (e.g., funder type, intervention type, masking, phase, ACT status, FDAAA period) as factors and entered them into the regression via indicator coding. To assess temporal changes, we conducted segmented regression using January 18, 2017, the Final Rule's effective date, as the breakpoint^15,30^. The dependent variable for all models was a composite AE reporting score (0-7), treated as continuous, with one point assigned for reporting (including zero values) in each of 7 required domains: (1) all-cause mortality, (2) number of participants with SAEs, (3) total number of SAE events (by system and study arm), (4) number of participants with OAEs occurring in ≥5% of participants, (5) total number of OAE events (≥5%), (6) number at risk for OAEs (≥5%), and (7) the specified OAE frequency threshold. Following previous studies, entries such as “0” or “reported, not calculated” were counted as reported, whereas nonmandated fields, such as the Medical Dictionary for Regulatory Activities (MedDRA), were excluded from the score^31,32^. The Durbin–Watson statistic indicated no significant autocorrelation in either the pre-2017 (2.036) or post-2017 (0.000) periods, supporting the validity of the time series analysis. Trends after 2020 were interpreted with caution because of smaller sample sizes. All analyses were conducted in R (v2024.09.1) and Python (v3.13.5), with significance defined as p < 0.05^33-35^.

### Risk of Bias and Confidence in Findings

Traditional methodological bias tools were not applied, as our study focused on the consistency of AE reporting rather than clinical outcomes. Instead, trials were classified by their reporting pattern as complete, partial, or discordant. Confidence in our findings is supported by our rigorous methodology, including systematic searches, dual independent data extraction, and careful matching of registry entries to publications.

### Protocol Registration, Amendments, and Deviations

The protocol was pre-registered with PROSPERO and posted to the OSF before data extraction, with all subsequent modifications and their rationales documented on OSF^36,37^.

## Results

### General Characteristics

Supplemental Table 1 summarizes the characteristics of the included TKA trials.

### Adverse Event Reporting Trends in ClinicalTrials.gov and Publications

AE reporting was consistently more complete in ClinicalTrials.gov than in publications (Table 1). Before the Final Rule, 47% (28/60) of trials reported SAEs in the registry, compared with 20% (12/60) in publications (p = 0.004). Mortality was the most omitted outcome; pre-Final Rule, 38% (23/60) of registries and 87% (52/60) of publications excluded it (p = 0.001). Post-Final Rule, only one registry omitted it vs. 78% (25/32) of publications (p = 0.001).

**TABLE I:** Adverse Event Reporting in Studies Before and After Final Rule Implementation

Adverse Event Domain	Study Start Before Final Rule (N = 60)			Study Start After Final Rule (N = 32)		
	ClinicalTrials.gov	Publication	p-value	ClinicalTrials.gov	Publication	p-value
No. of studies reporting AEs, by type						
Serious adverse events	28 (47%)	12 (20%)	**0.004** *	15 (47%)	10 (31%)	0.305*
Other adverse events	29 (48%)	23 (38%)	0.357*	14 (44%)	11 (34%)	0.608*
Deaths	37 (62%)	8 (13%)	**<0.001** *	31 (97%)	7 (22%)	**<0.001** *
Studies reporting zero AE, by type						
Serious adverse events	7 (12%)	10 (17%)	0.601*	2 (6%)	4 (12%)	0.672*
Other adverse events	14 (23%)	8 (13%)	0.238*	4 (12%)	3 (9%)	1.000*
Deaths	31 (52%)	4 (7%)	**<0.001** *	28 (88%)	4 (12%)	**<0.001** *
Studies not reporting AEs, by type						
Serious adverse events	32 (53%)	43 (72%)	0.059*	17 (53%)	21 (66%)	0.445*
Other adverse events	31 (52%)	31 (52%)	1.000*	18 (56%)	15 (47%)	0.617*
Deaths	23 (38%)	52 (87%)	**<0.001** *	1 (3%)	25 (78%)	**<0.001** *
Median no. of AE per trial (interquartile range/range)						
Serious adverse events	4.5 (0.8-14.0/0-314)	0 (0.0-0.0/0-30)	**0.002** †	4 (1.5-14.0/0-58)	3.5 (0.0-12.5/0-29)	0.450†
Other adverse events	1 (0.0-34.0/0-156)	5 (0.0-20.5/0-635)	0.455†	13.5 (0.2-82.2/0-250)	3 (1.0-9.0/0-107)	0.406†
Deaths	0 (0.0-0.0/0-4)	0.5 (0.0-1.2/0-4)	**0.041** †	0 (0.0-0.0/0-1)	0 (0.0-1.0/0-1)	**0.034** †
Median no. of affected patients per trial (interquartile range/range)						
Serious adverse events	0 (0.0-5.0/0-98)	0 (0.0-0.0/0-15)	0.077†	0.5 (0.0-4.2/0-44)	3.5 (0.0-11.8/0-31)	0.425†
Other adverse events	0 (0.0-8.5/0-266)	3.5 (0.0-7.8/0-208)	0.264†	0 (0.0-28.5/0-146)	6 (2.0-76.0/0-223)	0.096†
Deaths	0 (0.0-0.0/0-4)	0.5 (0.0-1.2/0-4)	**0.041** †	0 (0.0-0.0/0-1)	0 (0.0-1.0/0-1)	**0.034** †

Additional number of Pre-Final Rule AE events reported in publications in alternate format (not per patient or per event): 5 SAEs, 6 OAEs, and 0 deaths.

Additional number of Post-Final Rule AE events reported in publications in alternate format (not per patient or per event): 1 SAEs, 6 OAEs, 0 deaths. Bold items were deemed statistically significant p-values.

AE = adverse event, OAE = other adverse event, and SAE = serious adverse event.

* Based on Chi-square or Fisher exact test.

† Based on Mann–Whitney *U* test.

‡Statistically significant difference (p < 0.05).

The median SAEs per trial was higher in the registry (4.5) than in publications (0) (p = 0.002).

### Concordance Between Registry and Publication

Concordance between registry and publication reporting of SAEs was poor, with 87% (80/92) of all trials showing mismatches in the number of patients affected (Table II). ClinicalTrials.gov reported more SAE-affected patients in 39% (36/92) of trials, while publications did so in only 3% (3/92). SAE event count mismatches occurred in 95% (87/92) of trials, with 32% (29/92) reporting more SAEs in the registry and 26% (24/92) in the publication. Notably, 38% (35/92) of studies failed to report SAE counts in either source. Following the implementation of the Final Rule, omission of SAE event counts in both sources declined from 47%, in pre-Final Rule trials deemed ACTs, to 33% in post-Final Rule FDA-regulated studies, showing modest improvement. AE reporting locations varied widely, and 19% of post-Final Rule FDA-regulated trials did not report AEs at all in their publications.

**TABLE II:** Concordance of SAE Reporting and Documentation of AE

	All Studies (n = 92)	FDA-Regulated Studies	
		Pre-Final Rule Start Date (n = 36)	Post-Final Rule Start Date (n = 27)
SAE patient count mismatch (ClinicalTrials.gov vs publication)			
No	12 (13%)	5 (14%)	5 (19%)
Yes	80 (87%)	31 (86%)	22 (81%)
More in publication	3 (3%)	1 (3%)	1 (4%)
More in registry	36 (39%)	10 (28%)	10 (37%)
Publication NR, registry 0	37 (40%)	18 (50%)	10 (37%)
SAE event count mismatch (ClinicalTrials.gov vs publication)			
No	5 (5%)	2 (6%)	2 (7%)
Yes	87 (95%)	34 (94%)	25 (93%)
More in publication	24 (26%)	20 (56%)	3 (11%)
More in registry	29 (32%)	8 (22%)	8 (30%)
Publication NR, registry 0	7 (8%)	5 (14%)	2 (7%)
Registry NR, publication 0	9 (10%)	3 (8%)	3 (11%)
Both NR	35 (38%)	17 (47%)	9 (33%)
Registry NR, publication RNC	3 (3%)	1 (3%)	1 (4%)
Location of AE reporting (ClinicalTrials.gov)			
AE table	90 (98%)	36 (100%)	25 (93%)
Outcome measures (listed AE as an outcome measure)	14 (15%)	5 (14%)	7 (26%)
Location of AE reporting (publication)			
Results (in text narrative)	56 (61%)	21 (58%)	22 (81%)
Table/figure	24 (26%)	7 (19%)	11 (41%)
Discussion	7 (8%)	3 (8%)	2 (7%)
Supplementary file	3 (3%)		
Not Reported	33 (36%)	13 (36%)	5 (19%)

AE = adverse event, NR = not reported, RNC = reported, not calculated, and SAE = serious adverse event.

### Comparative Analysis of SAE Reporting Across Registries and Publications

Supplemental Figure 1 presents a Bland-Altman plot comparing the number of SAEs reported in ClinicalTrials.gov with those in corresponding publications across trials which provided data in both sources. The majority of trials fell within the 95% confidence interval.

### Visual Distribution of SAE Rates by Enrollment

**Fig. 2:**
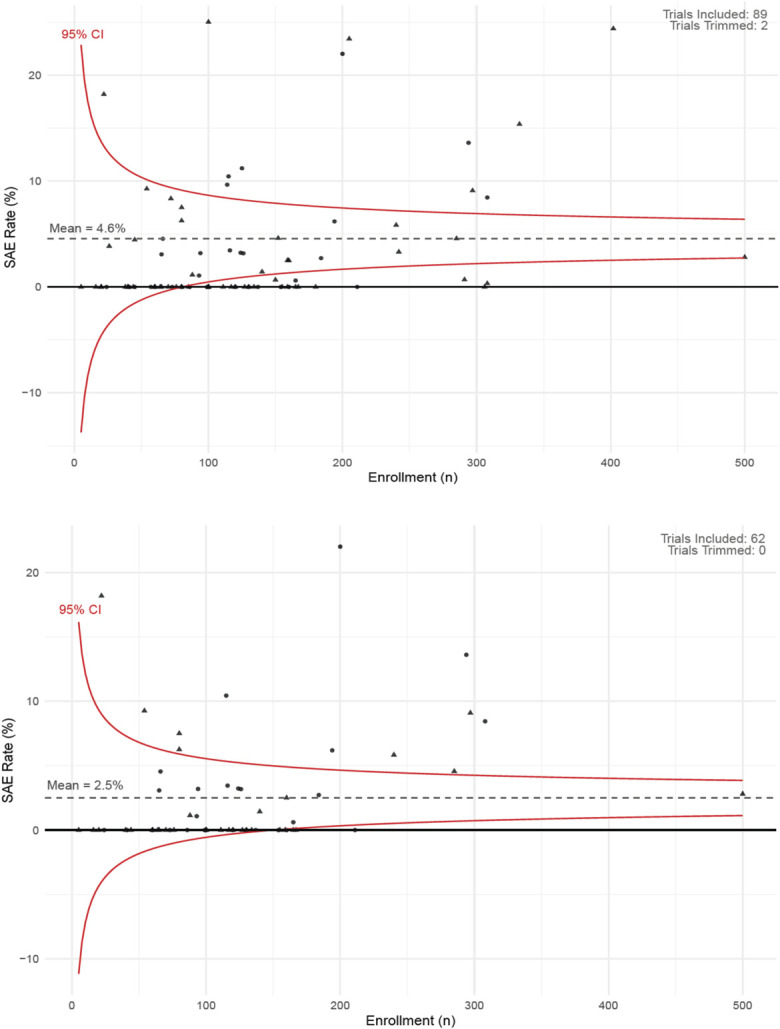
**Fig. 2-A** The funnel plot displays the reported SAE rates (% of affected participants) against trial enrollment size (n) for 89 included trials. The horizontal dotted line represents the mean SAE rate with solid lines indicating the 95% confidence interval (CI). Triangles represent clinical trials initiated pre-Final Rule. Circles represent studies initiated post-Final Rule. The distribution reflects heterogeneity in SAE reporting that may not be due to chance. **Fig. 2-B** The funnel plot displays the reported SAE rates (% of affected participants) against trial enrollment size (n) for 62 included likely applicable clinical trials. The horizontal dotted line represents the mean SAE rate with solid lines indicating the 95% CI. Two were trimmed to enhance the symmetry and visual presentation of the figure. Trials are categorized by the regulatory period. Triangles represent studies started pre-Final Rule. Circles represent trials started post-Final Rule. The distribution reflects heterogeneity in SAE reporting that may not be due to chance. SAE = serious adverse event.

Figure 2-A includes a total of 89 trials, with 2 trimmed as outliers and one excluded for missing SAE data. The mean SAE rate was 4.6%, but many small trials (N < 150) reported zero SAEs or rates outside the 95% confidence interval. Elevated rates may reflect true intervention risks or factors like high-risk populations, vague SAE definitions, or longer follow-up, while unusually low rates may suggest selective reporting or inconsistent data capture. No clear difference in SAE rates emerged between pre- and post-Final Rule trials. Among ACTs only (Fig. 2-B), the mean SAE rate was lower at 2.5%, with most trials falling within expected limits. However, 12 ACTs exceeded the upper confidence bound, potentially reflecting higher-risk interventions or improved event detection.

### Regression Analysis of Predictors and Policy-Driven Changes in AE Reporting

Larger trials, based on enrollment size, were significantly associated with more complete AE reporting (estimate = 0.003, p = 0.027). Additional model details are provided in Supplemental Table II.

**Fig. 3:**
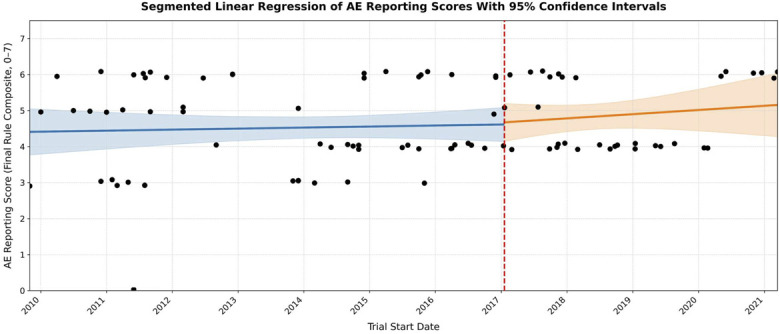
Segmented linear regression of AE reporting scores over time, stratified by trial start date (x-axis). The vertical dashed line marks the implementation of the FDAAA Final Rule on January 18, 2017. Each black dot represents the AE reporting score of an individual trial. Regression lines with 95% confidence intervals (shaded) depict trends in reporting scores before and after the Final Rule implementation. AE = adverse event, and FDAAA = Food and Drug Administration ACT.

Segmented regression revealed no significant change in AE reporting scores at the time of Final Rule implementation (Fig. 3). Overlapping confidence intervals suggest that observed differences are more likely attributable to random variability than to the policy itself.

### Trial-Level AE and SAE Categorization

A comprehensive summary of each trial's registry-reported AE and SAE categories and publication-reported AEs mapped to these categories is provided in Supplemental Table 3.

## Discussion

Our findings reveal a major gap in AE reporting, with ClinicalTrials.gov data often more complete than corresponding publications. Despite mandates like FDAAA 801, the 2017 Final Rule, and CONSORT Harms, discrepancies persist in reporting SAEs, OAEs, and deaths^38,39^. The Final Rule did not significantly improve reporting, underscoring a disconnect between regulatory requirements and publication practices and reinforcing broader concerns about the reliability of published AE data^3,4,6,8-13,18,26,40-46^.

Incomplete AE reporting is well documented in orthopaedics. A 2024 analysis of RCTs cited as supporting evidence for the AAOS clavicle fracture CPG reported an average of 9.32 appropriately reported items of the 18 CONSORT Extension for Harms items^42^. Similar studies of trials informing CPG for rotator cuff repair, anterior cruciate ligament reconstruction, hip fracture, and osteoarthritis also showed low adherence (22%-45%), with fewer than 50% of trials reporting even half the checklist items^5-8^. Terminology used to describe AEs is inconsistent as well: Mercer et al. identified 572 unique terms, across 117 studies, used to describe AEs, with 53 different labels used to describe fracture events alone^46^.

Our review of 92 TKA trials confirmed and amplified these trends: 95% had at least one SAE event count discrepancy, 87% differed in the number of patients affected, and mortality data were omitted from 78% of post-Final Rule publications despite appearing on ClinicalTrials.gov. Such gaps are not unique to TKA but reflect a systemic failure to prioritize transparent AE reporting within orthopaedics, where omissions risk diminished patient safety, device evaluation, regulatory decisions, and informed consent.

AE data from RCTs directly inform AAOS CPGs, FDA approval, and the risk-benefit assessments that guide surgical recommendations. Underreporting of AEs, including SAEs, can overstate safety, shaping recommendations based on incomplete evidence. AAOS CPGs, for example, are graded on risk-benefit profiles; the guideline for patellar resurfacing carries a strong recommendation (5/5 stars) based on studies in which “benefits of the recommended approach clearly exceed the potential harm”^47^. Inconsistent harms reporting (trials reporting only predefined subsets of AEs, vague composites like the “sum of the score of adverse effects,” lacking sufficient information to determine event counts, or listing multiple SAEs in the registry but none in their publication) can directly influence guideline strength and implementation^48-50^.

Incomplete AE reporting also hinders FDA review, where accurate event counts are essential to detect device-related safety signals. Devices without a previously approved “significantly equivalent” predicate undergo premarket approval (PMA) with mandatory reporting of any “death, serious injury, or device malfunction,” whereas equivalent devices may follow a faster premarket notification pathway with limited premarket AE reporting^51,52^. Poor reporting increases the risk that unsafe devices reach the market, as illustrated by metal-on-metal hip implants, which were cleared through equivalence determinations only to be withdrawn after widespread AE reports^53^. These gaps distort the perceived safety of TKA, influence shared decision-making, shape expectations of “acceptable” complication rates, and promote adoption of techniques or implants whose harms are incompletely understood. By highlighting these discrepancies, our study demonstrates how reliance on underreported literature can misinform surgeons, undermine patient counseling, and ultimately impact patient safety.

Several factors may account for discrepancies between AE data reported in registries and corresponding publications. Editorial constraints, such as word limits and expectations for concise presentation, may incentivize authors to streamline harms reporting, leading to the omission of complete AE tables or the exclusion of infrequent or seemingly minor events. Selective nonreporting can also occur when AE patterns appear unfavorable to the intervention or complicate the narrative of safety or efficacy. Inconsistent terminology or variable thresholds for defining AEs vs. SAEs can contribute to misclassification. Our inability to create a consistent cross-trial classification system underscores substantial variability in AE reporting. Lack of standardized terminology, inconsistent categorization, and incomplete reporting limit reproducibility and constrain understanding of established AEs.

Journals and editorial teams play a central role in improving AE reporting: requiring routine submission of the CONSORT Harms checklist, encouraging use of standardized terminology such as MedDRA, mandating submission of complete AE data sets as supplementary material, and tasking reviewers with verifying concordance between registry entries and manuscript data may help establish a more complete AE reporting landscape^54^. Integrating harms-specific prompts into peer-review forms, flagging omissions during editorial triage, and ensuring discrepancies are resolved before acceptance can create consistent expectations for complete harms reporting and produce a literature that more accurately reflects the risk profiles relied on by surgeons.

Strengths of our study include a pre-registered protocol, PRISMA adherence, and masked, duplicate data extraction to reduce bias. We examined both FDA-regulated and nonregulated trials, enabling a broader assessment. However, our exclusive reliance on ClinicalTrials.gov naturally limits how broadly these findings can be applied. Many orthopaedic device trials, especially those involving implants, are conducted internationally, often because of strict PMA requirements in the United States, and may be registered on platforms outside of ClinicalTrials.gov. These registries often lack standardized AE fields or do not require structured harms reporting. As a result, our data set likely overrepresents trials with a US regulatory focus and may not fully reflect global AE reporting practices. This limitation may reduce the applicability of our findings to international TKA research, where registry requirements, device regulations, and reporting standards can vary considerably.

This study reveals discrepancies in AE reporting in TKA trials, particularly for SAEs and deaths, despite regulatory mandates and reporting guidelines. These gaps hinder transparency, compromise risk-benefit assessments, and may affect surgical decisions and patient outcomes. Stricter adherence to reporting standards, stronger editorial oversight, and active enforcement are necessary to establish complete AE reporting practices essential to ethical research and a trustworthy evidence base. Future efforts must bridge the gap between regulatory expectations and actual practice.

## Appendix

Supporting material provided by the authors is posted with the online version of this article as a data supplement at jbjs.org (http://links.lww.com/JBJSOA/B106). This content was not copyedited or verified by JBJS.
